# Supplementary material for: Dosing of thromboprophylaxis and mortality in critically ill COVID-19 patients
Source: Crit Care. 2020 Nov 23;24:653. doi: 10.1186/s13054-020-03375-7 (PMC7680989; doi:10.1186/s13054-020-03375-7)
Supplement: Supplementary file 1 — Additional file 1. Local guidelines. [file 13054_2020_3375_MOESM1_ESM.docx]

# Local guidelines

In this document the guidelines used in both ICUs are translated to English. The two ICUs studied are normally one surgical ICU and one medical but during the pandemic both care for COVID-19 patients. When the medical ICU decided to use double dose thromboprophylaxis they used the guidelines from Karolinska University Hospital.

**Anesthesia and Intensive care**

| Administrative dept: | | Unit: |
| --- | --- | --- |
| Dept of Anesthesia and intensive care | | **Surgical ICU** |
| Dokument type: | | Dokument no: |
| **Guideline** | | 000474 |
| Document name: | | |
| **Covid-19 – airways, intubation, and treatment. Patients in ICU with suspicious or confirmed infection** | | |
| Created | Developed by: | |
| 032720 | Marianne Mörrby Ramberg, Brian Cleaver | |
| Last revision: | Revised by: | |
| 041620 | Marianne Mörrby Ramberg, Wolfram Johnen | |
| Printed on: | Approved by dept chief | |
| 041620 | Emma Jerkegren-Olsson | |

| **[…]** 032720: Thromboprophylaxis with Innohep® 4500 U once daily to patients with no contraindication.  Revision 200408: Patients with COVID-19 appear to have an increased risk for thromboembolic complications. Therefore, thromboprophylaxis dose should be given twice daily to patients with no contraindications.  Revision 200416: Due to high frequency of thromboembolic complications among critically ill COVID-19 patients we have decided to increase the dose of thromboprophylaxis. There is no strong evidence for this treatment decision, continuous evaluation will be performed and the treatment regime may change. If no contraindications or tendency of bleeding exists critically ill COVID-19 patients will be prescribed Innohep® 100 U/kg twice daily. **[…]** |
| --- |


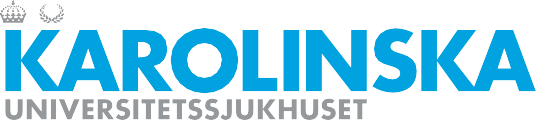


| Author:  Anders Oldner, Lars I Eriksson, Christina Agvald-Öhman | Pages: 7 |
| --- | --- |
|  | Dok-ID: Kar2-17857 |
| Approved by:  Björn Persson/Karolinska/SLL | Created: April 2020 |
|  | Valid thru: 2021-07-09 |

Funktion PMI – Perioperative Medicine and Intensive care

Pharmacological treatment of Covid-19 in Intensive care

**[…]**

Currently there is no evidence-based foundation to guide thromboprophylaxis dosage for critically ill Covid-19 patients. Until more evidence or information regarding treatment strategies is made available the following doses are recommended for **thromboprophylaxis**:

Weight:

50-90 kg Fragmin® 5000 IU twice daily, or equivalent

<50 kg Fragmin® 2500 IU twice daily, or equivalent

>90 kg Fragmin® 7500 IU twice daily, or equivalent

The dose may need adjustment with regard to degree of illness and tendency to bleed

**[…]**
