# Supplementary material for: Dosing of thromboprophylaxis and mortality in critically ill COVID-19 patients
Source: Crit Care. 2020 Nov 23;24:653. doi: 10.1186/s13054-020-03375-7 (PMC7680989; doi:10.1186/s13054-020-03375-7)
Supplement: Supplementary file 2 — Additional file 2. Flow chart. [file 13054_2020_3375_MOESM2_ESM.docx]

**165** patients admitted to ICU between March 6 and April 30 due to critical illness caused by laboratory confirmed COVID-19

**13** patients met exclusion criteria:

**5** had a short ICU length of stay

**4** had ongoing anticoagulant therapy due to thromboembolism diagnosed prior to ICU

**4** had no initial thromboprophylaxis in the ICU

**67** patients with low dose thromboprophylaxis

**152** patients got the dosing strategy of thromboprophylaxis following local recommendations for the time period they were admitted

**37** patients with high dose thromboprophylaxis

**48** patients with medium dose thromboprophylaxis
