# Supplementary material for: Dosing of thromboprophylaxis and mortality in critically ill COVID-19 patients
Source: Crit Care. 2020 Nov 23;24:653. doi: 10.1186/s13054-020-03375-7 (PMC7680989; doi:10.1186/s13054-020-03375-7)
Supplement: Supplementary file 3 — Additional file 3. Time varying effect. [file 13054_2020_3375_MOESM3_ESM.docx]

**Table 4. The risk of death when follow-up was split at first week**

Risk of death during the first 28 days, with follow-up split at the first week, among 152 patients admitted to the intensive care unit due to COVID-19 at Södersjukhuset, Stockholm, March 6 to April 30, 2020, by initial treatment regime with tinzaparin/dalteparin as thromboprophylaxis.

|  | |  |  |  | HR (95% CI) of death ≤28 days | | |
| --- | --- | --- | --- | --- | --- | --- | --- |
|  | |  |  |  |  |  |  |
| Initial dosing strategy of thromboprophylaxis | | No. of  patients | Events /  person-days | IR per 1.000 person-days  (95% CI) | Univariable  model | Multivariable model^1^ | Multivariable complete case model^2^ |
| Day 7 – 28^4^ | |  |  |  |  |  |  |
|  | High dose^5^ | 33 | 1 / 676 | 1.5 (0.2 – 10.5) | 0.09 (0.01 – 0.64) | 0.08 (0.01 – 0.62) | 0.07 (0.01 – 0.53) |
|  | Medium dose^6^ | 44 | 8 / 861 | 9.3 (4.6 – 18.6) | 0.53 (0.23 – 1.23) | 0.75 (0.32 – 1.78) | 0.63 (0.25 – 1.55) |
|  | Low dose^7^ | 59 | 18 / 1006 | 17.9 (11.3 – 28.4) | 1.00 (Ref.) | 1.00 (Ref.) | 1.00 (Ref.) |
|  |  |  |  |  |  |  |  |
| Day 0 – 7^4^ | |  |  |  |  |  |  |
|  | High dose^5^ | 37 | 4 / 247 | 16.2 (6.1 – 43.1) | 1.00 (0.25 – 4.00) | 0.85 (0.21 – 3.45) | 1.00 (N/A) |
|  | Medium dose^6^ | 48 | 4 / 321 | 12.5 (4.7 – 33.2) | 0.77 (N/A) | 0.94 (N/A) | 1.25 (0.30 – 5.22) |
|  | Low dose^7^ | 67 | 8 / 447 | 17.9 (9.0 – 35.8) | 1.11 (0.33 – 3.68) | 0.76 (0.22 – 2.62) | 0.90 (0.26 – 3.17) |

CI indicates Confidence Interval; IR, Incidence Rate; HR, Hazard Ratio.

^1^ Adjusted for sex, age (continuously), body-mass index (</≥30 kg/m^2^ and missing [n=6]), invasive respiratory support (yes/no), and simplified acute physiology score III (continuously)

^2^ Similar adjustments as in the multivariable model but complete case analysis with BMI flexibly modeled with restricted cubic splines at three knots over the 10^th^, 50^th^, and 90^th^, percentile of the distribution of body-mass index in the population.

^3^ Similar adjustments as in the multivariable complete case model but with body-mass index imputed due to missing values (n=6).

^4^ Follow-up time was split after 7 days and the model was fitted with an interaction between thromboprofylactic strategy and follow-up time (</≥7 days). *P* for interaction with time = 0.17.

^5^ tinzaparin, ≥175 IU/kg of body weight OD, or dalteparin, ≥200 IU/kg of body weight OD

^6^ tinzaparin, >4500 IU OD to <175 IU/kg of body weight OD, or dalteparin, >5000 IU OD to <200 IU/kg of body weight OD

^7^ tinzaparin, 2500-4500 IU OD, or dalteparin, 2500-5000 IU OD
