# Supplementary material for: Dosing of thromboprophylaxis and mortality in critically ill COVID-19 patients
Source: Crit Care. 2020 Nov 23;24:653. doi: 10.1186/s13054-020-03375-7 (PMC7680989; doi:10.1186/s13054-020-03375-7)
Supplement: Supplementary file 4 — Additional file 4. Sensitivity analyses. [file 13054_2020_3375_MOESM4_ESM.docx]

**Risk of death by initial dosing strategy of thromboprophylaxis when adjusting for treatment with glucocorticoids, when excluding patients with decreased dose compered to initial dose and when excluding patients who had any change in dose**

Risk of death during the first 28 days among 152 patients admitted to the intensive care unit due to COVID-19 at Södersjukhuset, Stockholm, March 6 to April 30, 2020, by initial dosing strategy with tinzaparin/dalteparin as thromboprophylaxis.

|  |  |  |  | HR (95% CI) of death ≤28 days | | |  |  |  |
| --- | --- | --- | --- | --- | --- | --- | --- | --- | --- |
|  |  |  |  |  |  |  |  |  |  |
| Initial dosing strategy of thromboprophylaxis | No. of  patients | Events /  person-days | IR per 1.000 person-days  (95% CI) | Univariable  model | Multivariable model^a^ | Multivariable imputed model^b^ | Multivariable model with glucocorticoids | Multivariable model without those who had a decreased dose compared to initial dose (n=147) | Multivariable model among those who had no change in dose (n=86) |
| High dose^c^ | 37 | 5 / 923 | 5.4 (2.3 – 13.0) | 0.31 (0.12 – 0.82) | 0.33 (0.13 – 0.87) | 0.30 (0.11 – 0.81) | 0.32 (0.12 – 0.85) | 0.34 (0.13 – 0.90) | 0.33 (0.11 – 1.00)^f^ |
| Medium dose^d^ | 48 | 12 / 1182 | 10.2 (5.8 – 17.9) | 0.59 (0.30 – 1.16) | 0.88 (0.43 – 1.83) | 0.87 (0.42 – 1.82) | 0.83 (0.39 – 1.73) | 0.90 (0.44 – 1.87) | 1.15 (0.38 – 3.47) |
| Low dose^e^ | 67 | 26 / 1453 | 17.9 (12.2 – 26.3) | 1.00 (Ref.) | 1.00 (Ref.) | 1.00 (Ref.) | 1.00 (Ref.) | 1.00 (Ref.) | 1.00 (Ref.) |

CI indicates Confidence Interval; IR, Incidence Rate; HR, Hazard Ratio.

^a^ Adjusted for sex, age (continuously), body-mass index (</≥30 kg/m^2^ and missing [n=6]), invasive mechanical ventilation (yes/no), and Simplified Acute Physiology Score III (continuously)

^b^ Adjusted like the multivariable model but with body-mass index imputed due to missing values (n=6), and flexibly modeled with restricted cubic splines at three knots over the percentile (10^th^, 50^th^, and 90^th^) distribution of body-mass index in the population.

^c^ tinzaparin, ≥175 IU/kg of body weight per OD, or dalteparin, ≥200 IU/kg of body weight OD

^d^ tinzaparin, >4500 IU OD to <175 IU/kg of body weight OD, or dalteparin, >5000 IU OD to <200 IU/kg of body weight OD

^e^ tinzaparin, 2500-4500 IU OD, or dalteparin, 2500-5000 IU OD

^f^ P = 0.049
