# Supplementary figures and images for: Dosing of thromboprophylaxis and mortality in critically ill COVID-19 patients
Source: Crit Care. 2020 Nov 23;24:653. doi: 10.1186/s13054-020-03375-7 (PMC7680989; doi:10.1186/s13054-020-03375-7)

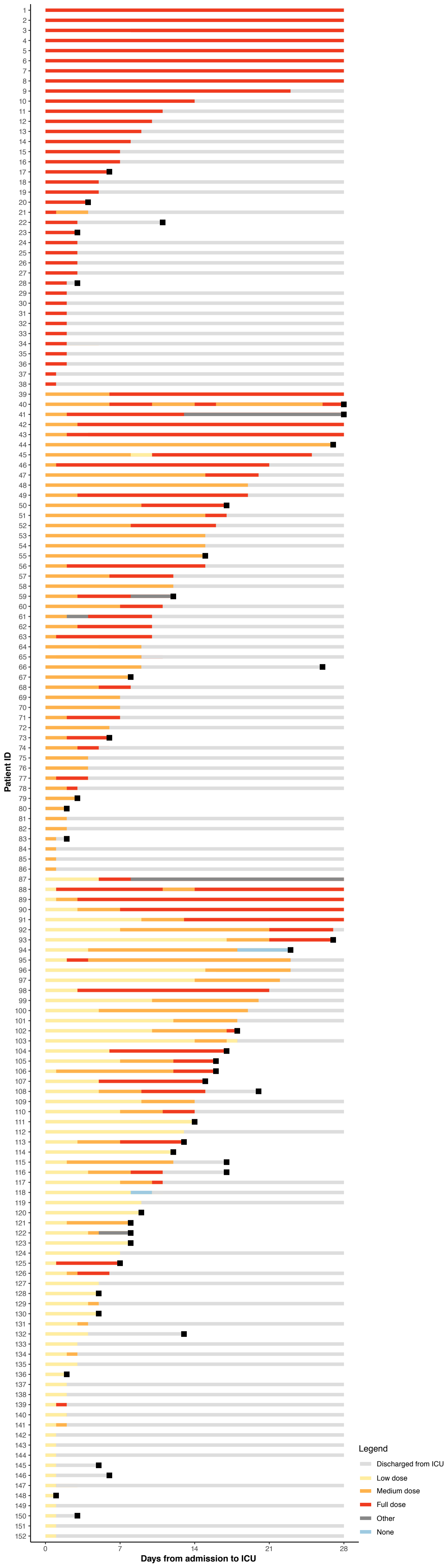

Supplement: Supplementary file 5 — Additional file 5. Individual does of thromboprophylaxis over time. [file 13054_2020_3375_MOESM5_ESM.pdf]
